# Several Human Cyclin-Dependent Kinase Inhibitors, Structurally Related to Roscovitine, As New Anti-Malarial Agents

**DOI:** 10.3390/molecules190915237

**Published:** 2014-09-23

**Authors:** Sandrine Houzé, Nha-Thu Hoang, Olivier Lozach, Jacques Le Bras, Laurent Meijer, Hervé Galons, Luc Demange

**Affiliations:** 1 Laboratoire de Parasitologie, CNR du Paludisme, AP-HP, Hôpital Bichat & UMR 216 IRD, Université Paris Descartes, Sorbonne Paris Cité, UFR des Sciences Pharmaceutiques, 4 avenue de l’Observatoire, Paris 75006, France; E-Mails: sandrine.houze@bch.aphp.fr (S.H.); jacques.lebras@bch.aphp.fr (J.L.B.); 2 Laboratoire de Chimie et Biochimie Pharmacologiques et Toxicologiques (LCBPT), UMR 8601 CNRS, Université Paris Descartes, Sorbonne Paris Cité, UFR Biomédicale des Saints Pères, 45 rue des Saints-Pères, Paris 75270, France; E-Mail: luc.demange@parisdescartes.fr; 3 Protein Phosphorylation and Human Diseases Group, CNRS, USR 3151, Station biologique, Roscoff 29680, France; E-Mails: olivier.lozach@sb-roscoff.fr (O.L.); meijer@sb-roscoff.fr (L.M.); 4 ManRos Therapeutics, Hôtel de Recherche, Centre de Perharidy, Roscoff 29680, France; E-Mail: herve.galons@parisdescartes.fr; 5 Laboratoire de Pharmacochimie, INSERM U 1022, Université Paris Descartes, Sorbonne Paris Cité, UFR des Sciences Pharmaceutiques, 4 avenue de l’Observatoire, Paris 75006, France; 6 Institut de Chimie de Nice (ICN), UMR 7272 CNRS, Université de Nice Sophia-Antipolis, Parc Valrose, Nice 06108, France

**Keywords:** *Plasmodium falciparum*, cyclin-dependent kinases CDKs, *Pf*CDKs, 2,6,9-trisubstituted purines, roscovitine, Buchwald-Hartwig amination

## Abstract

In Africa, malaria kills one child each minute. It is also responsible for about one million deaths worldwide each year. *Plasmodium falciparum*, is the protozoan responsible for the most lethal form of the disease, with resistance developing against the available anti-malarial drugs. Among newly proposed anti-malaria targets, are the *P. falciparum* cyclin-dependent kinases (*Pf*CDKs). There are involved in different stages of the protozoan growth and development but share high sequence homology with human cyclin-dependent kinases (CDKs). We previously reported the synthesis of CDKs inhibitors that are structurally-related to (*R*)-roscovitine, a 2,6,9-trisubstituted purine, and they showed activity against neuronal diseases and cancers. In this report, we describe the synthesis and the characterization of new CDK inhibitors, active in reducing the *in vitro* growth of *P. falciparum* (3D7 and 7G8 strains). Six compounds are more potent inhibitors than roscovitine, and three exhibited IC_50_ values close to 1 µM for both 3D7 and 7G8 strains. Although, such molecules do inhibit *P. falciparum* growth, they require further studies to improve their selectivity for *Pf*CDKs.

## 1. Introduction

Malaria remains today one of the most devastating infectious diseases in the world. Despite remarkable progress in the global fight against malaria, this deadly protozoan infection takes an estimated 700,000 lives per year, mostly African children under five years of age [1].

Among the different species of protozoan parasites responsible for malaria, *Plasmodium falciparum*, which is transmitted to humans through the bite of infected *Anopheles* mosquitoes, is the most lethal [2]. Current treatments effective for malaria include chloroquine, mefloquine and artemisinin, but these drugs becoming less effective due to the gradual emergence of drug-resistant strains [3]. Artemisinin-based combination therapies have been adopted as the first-line antimalarial agents of choice against these resistant *Plasmodium* parasites. Recently, however, there has been increasing concern regarding the development of resistance to the artemisinins further emphasizing the need for new antimalarial agents with different mechanisms of action [4]. As a consequence of the resistance, an alarming resurgence of malaria has occurred, and the anti-malarial drug space should be urgently extended. New targets, such as the apicoplast [5], the protozoan proteases [6], or the specific mitochondrial electron transport chain are currently being investigated [7]. Protein kinases, which regulate protozoan growth and differentiation during its life cycle, have also emerged to be among the most promising new anti-malarial targets [8,9,10,11]. A short survey of the recent literature highlights the success of targeting *P. falciparum* kinases such as thymidinate kinase (K_I_ = 20 µM) [12], cGMP-dependent protein kinase (IC_50_ = 8 nM) [13], calcium-dependent kinase 1 (IC_50_ in the 10–20 nM range) [14,15], and *Pf*RIO-2 kinase, which regulates the plasmodial ribosome biogenesis [16].

Among the human kinome, the cyclin-dependent kinases (CDKs) constitute a family of 20 ubiquitous serine/threonine kinases [17,18,19]. They are mainly involved in cell-cycle regulation [20,21], apoptosis [22] and gene transcription [23]. Importantly, regulatory proteins called cyclins form dimeric structures with the CDKs and are therefore responsible for their activation [17]. In humans, CDKs deregulation is involved in severe pathologies such as cancers (solid tumors and leukemias) [18], neuronal disorders (including Alzheimer’s and Parkinson’s diseases) [24,25] and type 2 diabetes [26]. Indeed, CDKs are considered as promising therapeutic targets in a wide range of pathologies including viral infections (AIDS), renal diseases (glomerulonephritis and polycystic kidney disease), inflammation and cardio-vascular diseases [27].

A family of *P. falciparum* kinases, *Pf*CDKs, which share high sequence homology with human CDKs, were identified in the 1990s. Thus, *Pf*PK5 (around 60% of sequence identity with human CDK1 and CDK5) [28], *Pf*PK6 (57% similarity with the catalytic domain of CDK2) [29], and *Pf*mrk (46% of sequence identity with CDK7) [30] have been extensively studied and characterized. Interestingly, CDKs and *Pf*CDKs exhibit similar activation by phosphorylation, catalyzed by CDK activating kinases [28], and interaction with endogenous inhibitors, such as p21^CIP1^ [31]. Moreover, *Pf*CDKs co-incubation with human cyclins leads to their functional activation [32]. Despite these strong molecular and functional homologies, HsCDKs and *Pf*CDK have structurally diverged to allow selective targeting the *Pf*CDKs without affecting host CDKs. Indeed, many known CDKs inhibitors are unable to inhibit *Pf*CDKs [9,10,11,12,33,34,35,36,37,38].

**Figure 1:**
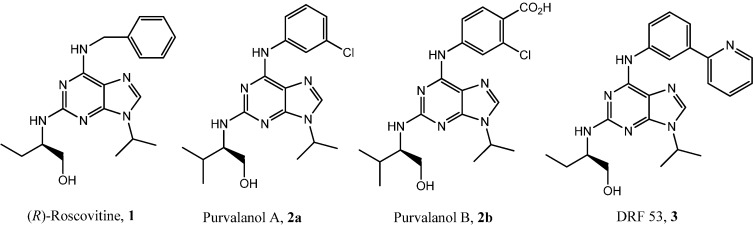
Chemical structures of some purine CDK inhibitors.

The purine scaffold is widely used for the development of therapeutic agents [39,40], and it has therefore provided several CDKs inhibitors such as (*R*)-roscovitine (**1**) or purvalanol A (**2a**) and B (**2b**) (Figure 1) [41]. Basically, (*R*)-roscovitine, a 2,6,9-trisubstituted purine, is a very promising molecule developed by Cyclacel Pharmaceuticals which reached phases 2 and 2b clinical trials against different types of cancers, and phase 1 clinical trial against glomerulonephritis. Interestingly, the (*R*)-roscovitine clinical trials pointed out the low cytotoxicity of this molecule. To summarize, a daily oral dose of 2 g was found as the maximal tolerated dose in human. Pharmacokinetics studies showed a rapid elimination of this drug with a half-life between 60 and 90 min. In humans, following oral administration, (*R*)-roscovitine offers good oral bioavailability and undergoes a rapid passage into the blood, distribution in tissues, and metabolism. Altogether, these results suggest that oral administration is possible for extended periods of treatment [41].

This molecule binds CDKs in the ATP pocket and is rather selective for CDK1, CDK2, CDK5, CDK7 and CDK9, but interacts with other kinases although with lower affinity (e.g., casein kinase 1 (CK1), dual specificity, tyrosine phosphorylation regulated kinases (DYRKs), pyridoxal kinase) [42,43].

In this context, our team focused its efforts on the identification of new CDK inhibitors structurally-related to (*R*)-roscovitine [44]. Thus, by means of innovative Buchwald-Hartwig aminations and Suzuki cross-coupling reactions, we developed several series of 2,6,9-trisubstituted purines including 6-aminoaryl, 6-aminoheteroaryl and 6-aminobiaryl moieties [45,46]. Biological characterization of these molecules revealed “hit” compounds such as DRF053 (**3**), one of the most potent CK1 inhibitor (IC_50_ = 14 nM) and CDK5 inhibitor (IC_50_ = 80 nM), which antagonizes amyloïd-β production in the N2A-APP_659_ cell model, in a dose-dependent manner [44]. In addition, introduction of 6-aminoheteroaryl moieties leads to inhibitors which are 5- to 10-fold more potent than (*R*)-roscovitine in cytotoxicity assays on human neuroblastoma SH-SY5Y cells (IC_50_ ranging from 1.8 µM to 2.1 µM) [45].

**Figure 2:**
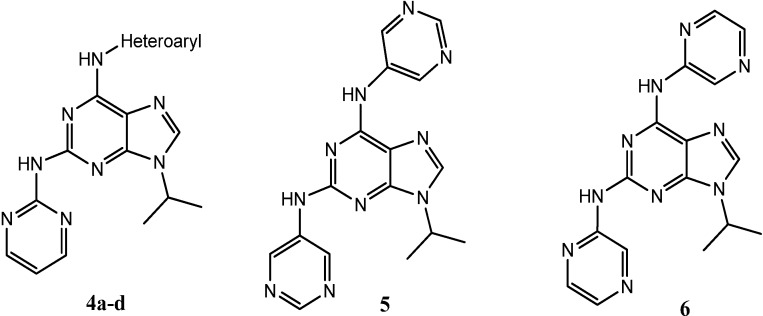
General structure for the newly-synthesized compounds.

In the present paper, we report the synthesis of a new series of 2,6,9-trisubstituted purines structurally-related to (*R*)-roscovitine, and bearing amino-heterocyclic motives in the C^2^ purine ring, such as 2-aminopyrimidine (**4a**–**d**) and 5-aminopyrimidine (**5**) mimicking the pyrimidine core found in the anti-cancer drug imatinib (Gleevec^®^), and an aminopyrazine (**6**) (Figure 2). In addition, we report the characterization of new 2,6,9-trisubstituted purines belonging to previously reported series of HsCDKs inhibitors. All these molecules were evaluated against a panel of human kinases including CDK1, CDK2, CDK5, glycogen synthase kinase-3 (GSK-3), CK1 and DYRK1A. Finally, these newly-synthesized molecules and several other compounds belonging to our library of purines were screened as potential growth inhibitors of *P. falciparum*.

## 2. Results and Discussion

### 2.1. Chemical Synthesis

#### 2.1.1. Synthesis of New Purines Series Bearing C^2^ Amino-Heterocyclic Moities

**Scheme 1:**
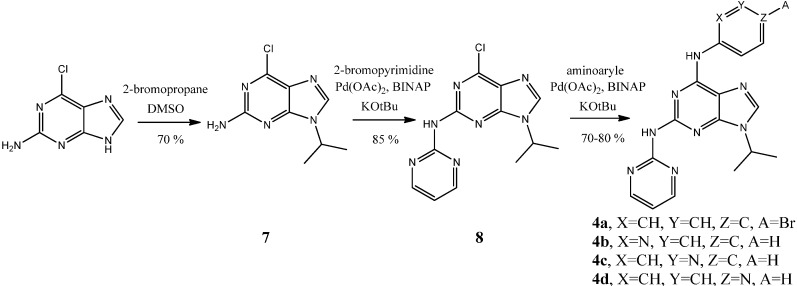
Synthetic route to compounds **4a**–**d**.

Compounds **4a**–**d** were synthesized following an efficient three-step procedure outlined in Scheme 1. Briefly, the commercially available 2-amino-6-chloropurine was regioselectively converted into 2-amino-6-chloro-9-*iso*-propylpurine (**7**) in 70% yield using 2-bromopropane at 15–18 °C in DMSO for 5 days. Then, compound **7** was reacted with 2-bromopyrimidine under the palladium catalysis Buchwald-Hartwig amination conditions we previously reported (Pd(OAc)_2_, 10%; BINAP 10%, KOtBu, 1.5 eq.) to afford 6-chloro-9-isopropyl-*N*-(pyrimidin-2-yl)-2-aminopurine (**8**) in 85% yield [47]. A second amination gave the final compounds **4a**–**c** with 70%–80% yield. Lower amount of catalyst and ligand (Pd(OAc)_2_, 4%; BINAP 6%, tBuOK, 1.5 eq.) were required for this second Buchwald-Hartwig amination to avoid polysubstitution on the purine scaffold [45,48].

We already reported the formation of compounds **5** and **6** as side products from a Buchwald-Hartwig amination on the 2,6-dichloro-9-isopropylpurine (**9**), obtained through a region selective N^9^ alkylation with the commercially available 2,6-dichloropurine [45].

**Scheme 2:**
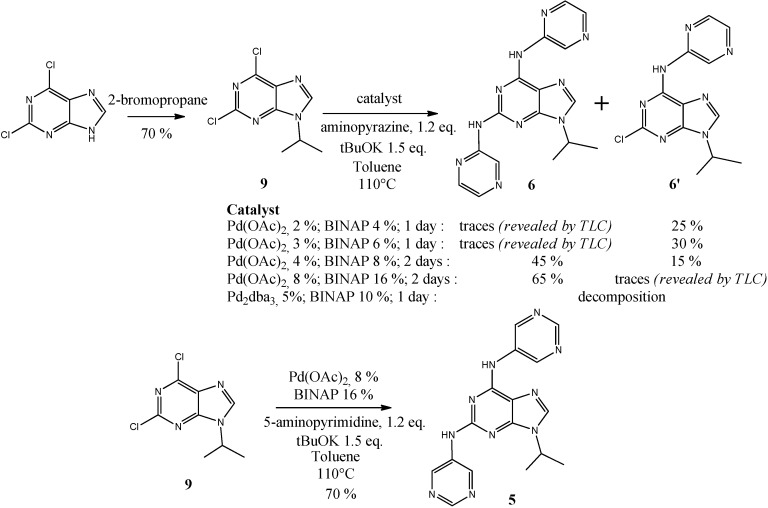
Synthetic route to compounds **5** and **6** and Buchwald-Hartwig diamination optimization.

We report here the optimization of this catalytic system in order to obtain exclusively this di-amination product in good yield. As shown in Scheme 2, we used aminopyrazine as a nucleophile for this optimization, and our study revealed that the use of 8% of Pd(OAc)_2_ pre-catalyst and 16% of racemic BINAP ligand gave compound **6** with the highest conversion rate. In contrast to our previous study on amination with aminopyridines, we do not observe here the formation of a triarylamine product based upon the aminopyridazine scaffold [47]. Surprisingly, the replacement of the Pd(II) pre-catalyst by a Pd(0) pre-catalyst (Pd_2_dba_3_) leads only to decomposition products. Finally the use of the Pd(OAc)_2_ catalytic system allowed the formation of compound **5** in 70% yield (Scheme 2).

#### 2.1.2. Synthesis of New 6-Aminobiaryl Molecules Structurally-Related to (*R*)-Roscovitine

**Scheme 3:**
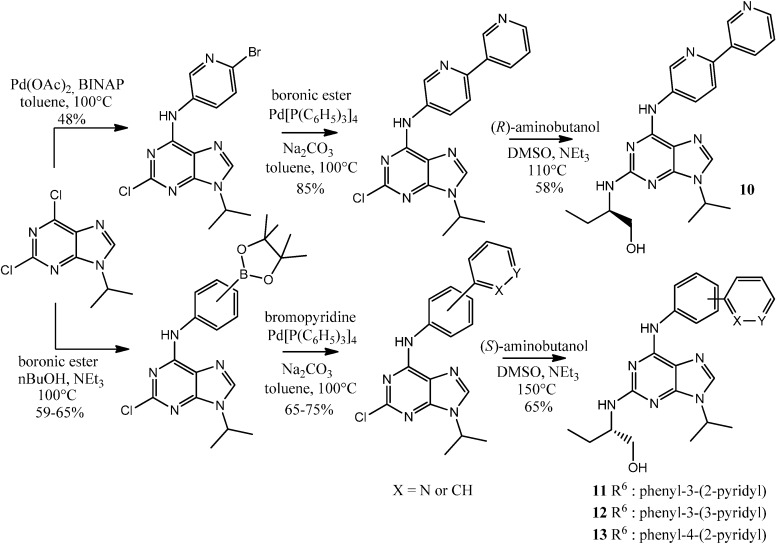
Synthesis of new 6-aminobiaryle **10**–**13**.

To complete our purine library with compounds structurally-related to (*R*)-roscovitine, we synthesized compounds **10**–**13** according to previously reported synthesis routes, whose key steps are Buchwald-Hartwig aminations and Suzuki cross-coupling reactions (Scheme 3) [44,47].

### 2.2. Molecular Assays on Mammalian Kinases

All newly synthesized compounds were tested as potential inhibitors on a panel of several mammalian kinases. The effects on CDK1/cyclin B, CDK2/cyclin A, CDK5/p25, GSK-3αβ, CK1δ/ε and DYRK1A kinase activity are reported as the IC_50_ values calculated from dose-response curves. They are summarized in Table 1. The inhibition was determined covering a range of concentrations of the compounds by means of a [γ-^33^P] assay as described in the experimental section.

Globally, compounds **4a**–**d**, with a 2-aminopyrimidine in the C^2^ position, and compound **6** with a 2-aminopyrazine in the C^2^ position, are poor kinase inhibitors when compared to (*R*)-roscovitine (**1**) or DRF053 (**3**). Moreover, we recently reported that **4a**–**d** analogs bearing an aminoalcohol in the purine C^2^ position inhibits CDKs with IC_50_ values ranging from 0.01 µM to 1.00 µM. Altogether, these results underline the relevance of the C^2^ aminoalcohol to improve the CDKs ATP-pocket binding, since the hydroxyl group of the amino-alcohol is engaged in a H-bonding network involving water molecules. Therefore this hydroxyl group plays a critical role for improved inhibitor binding [49,50].

**Table 1:** Newly-synthesized trisubstituted purines effects on several mammalian protein kinases activity. Purine derivatives were tested at various concentrations as described in the experimental section; IC_50_ values are provided in µM were estimated, dose-response curves and are expressed in µM. All assays were run in triplicates and data points were within less than a 10% range.

Compounds	IC_50_ (µM) ^a^					
	CDK1	CDK2	CDK5	GSK-3αβ	CK1δ/ε	DYRK1A
**1**	0.35	0.70	0.2	N.I.	2.3	-
**3**	0.22	-	0.08	>10	0.01	-
**4a**	-	-	1.0	1.0	1.0	3.0
**4b**	12	-	>10	>10	>10	8
**4c**	3.0	1.8	4.3	>10	>10	8
**4d**	9.3	-	7.0	>10	2.8	5.2
**5**	-	0.2	0.4	>10	0.2	0.5
**6**	-	2.8	13	>10	1.1	0.5
**10**	0.01	-	0.03	>10	0.2	-
**11**	0.8	-	0.4	>10	0.04	-
**12**	0.2	-	0.1	>10	0.2	-
**13**	0.2	0.2	-	>10	0.5	-

^a^ IC_50_ value reported as >10 indicates that the compound display no activity at the highest concentration tested (10 µM); -: not tested.

The influence of the amino-alcohol stereochemistry on the kinase inhibition appeared to be limited. Nevertheless, as reported on several series of 2,6,9-trisubstituted purines including (*R*)-roscovitine itself, the (*R*) stereoisomer showed a higher affinity for CDK than the (*S*) one. This might be highlighted on CDK5 by comparing **11** (IC_50_ = 0.4 µM) and **12** (IC_50_ = 0.1 µM) with their corresponding (*R*) stereoisomers (IC_50_ = 0.08 µM and IC_50_ = 0.05 µM, respectively [44]).

As expected, compound **10** is a potent CDK5 inhibitor (IC_50_ = 0.03 µM). This confirms the potency of the C^6^ biaryl moiety in term of H-bonding and molecule stacking [44], since its IC_50_ value is close to previous results obtained on CDK5 with the 6-aminobiaryl series of inhibitors. For the drug discovery field, CDK5 is a very promising target. Indeed, this kinase is involved in multiple neuronal activities (neuronal survival and migration), development of the cerebral cortex, phosphorylation of tau and production of β-amyloïd, pain signaling and pancreatic secretion of insulin [25,51,52,53]. To date, CDK5 specific inhibitors reported by others include complex scaffolds such as pyrazolopyrimidines (IC_50_ = 0.03 µM) [54], 2,4-diaminothiazoles (IC_50_ values included between 0.015 µM and 1 µM) [55] and cyclohexyl-thiophene moieties (IC_50_ values included between 0.035 µM and 1 µM) [56].

Compound **11** is a potent inhibitor of CK1, with an IC_50_ value of 0.04 µM. This kinase is involved in multiple physiological events, such as circadian rhythm regulation, and its implication in Alzheimer’s disease was strongly suggested [43,57]. With the significant exception of our previously reported series or trisubstituted purines including DRF053 [34], known CK1 inhibitors encompass generally polycyclic complex structures, including natural products such as hymenialdisine (IC_50_ = 0.03 µM) [58], a pyrrole-imidazole alkaloid extracted from marine sponges, and original synthetic molecules [59].

Interestingly, compounds **5** and **6** retain a marked inhibitory effect on DYRK1A. This activity is very close to the IC_50_ (ranging from 0.21 µM to 0.48 µM) that we previously reported for this kinase with other 2,6,9-trisubstituted purines [45]. Moreover this inhibition is stronger compared to that of (*R*)-roscovitine (87% of remaining DYRK1A kinase activity at 1 µM) and of purvalanol A (88% of remaining DYRK1A kinase activity at 0.1 µM) [43]. This is important because DYRK1A deregulation is involved in severe neurodegenerative pathologies, such as Down’s syndrome and the cognitive deficits associated with Alzheimer’s disease [60,61]. Only a few sub-micromolar DYRK1A inhibitors have been described, such as leucettines (IC_50_ values around 0.04 µM) [62,63], lamellarin D derivatives (IC_50_ = 0.07 µM) [64] or acridone alkaloids from *Glycosomis chlorosperma* (IC_50_ = 0.075 µM) [65], and synthetic original molecules, such as 4,7-disubstituted pyrido[3,2d]pyrimidines [66], 6-arylquinazolin-4-amines [67] and phtalazinone [68]. Nevertheless, these molecules have complex organic structures requiring multi-steps synthesis and their “hit to lead” optimization might be difficult. Finally, our results confirm the potency of the purine scaffold as a potent initial scaffold to develop and optimize new DYRK1A inhibitors.

### 2.3. Evaluation on P. falciparum

**Figure 3:**
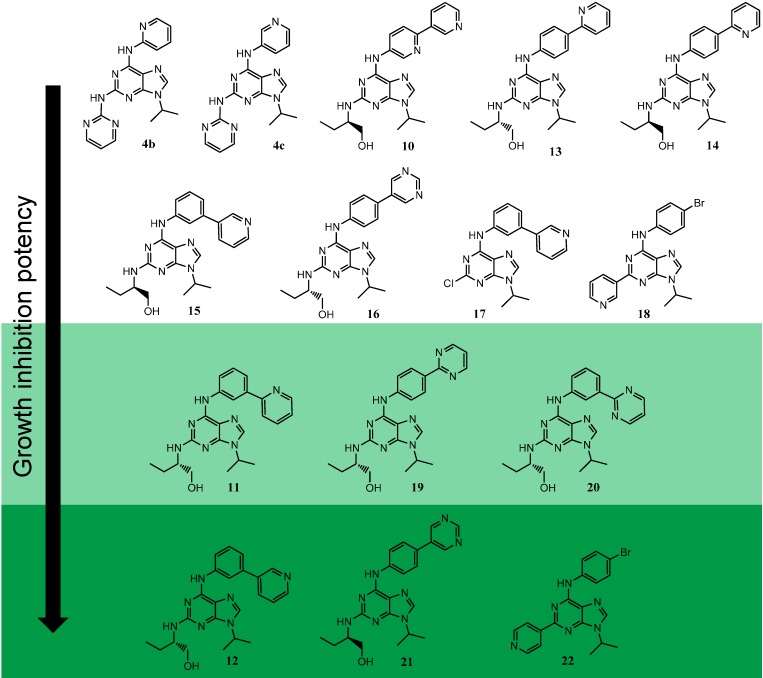
Structures of the 2,6,9-trisubstituted purines screened as *P. falciparum* growth inhibitors ^a^.

^a^ Inactive compounds are drawn on a white background; these molecules were not selected for further IC_50_ measurement. Compounds with IC_50_ > 6 µM on *Pf*3D7 are drawn with light grey background. The most potent *P. falciparum* growth inhibitors are drawn with dark grey background.

A selection of 15 molecules **4b**–**c**, **10**–**13** representing the different 2,6,9-trisubstituted purine series, including newly synthesized products was screened as anti-malarial agents on two different *P. falciparum* strains, *Pf*3D7, a standard drug-sensitive African stain, and *Pf*7G8, a brazilian chloroquino-resistant strain (Figure 3). The compound activity was evaluated during the parasitic erythocytic cycle, by the means of a [^3^H] hypoxanthine incorporation assay [69]. It is directly correlated to parasite viability, as described in the experimental section. IC_50_ values on mammalian kinases for the *P. falciparum* screened compounds are summarized in supplementary material, Table A1, appendix section.

At first, the activity of the fifteen compounds was screened at three different concentrations (10, 50 and 100 µM) on *Pf*3D7. Chloroquine which is not a kinase inhibitor, but which is the antimalarial reference drug was used as the positive control. This allowed the selection of the six most active compounds, which totally inhibit the parasite proliferation during its erythrocytic cycle at a concentration of 100 µM. Then, in a second assay, these six molecules plus (*R*)-roscovitine (**1**) and purvalanol A (**2a**) were evaluated in a dose-dependent assay, in order to determine their IC_50_ values on the two *P. falciparum* strains. Chloroquine was again used as a positive control.

As expected, each *P. falciparum* strain growth is partly inhibited by (*R*)-roscovitine and purvalanol A, and the latter exhibited the strongest activities. Nevertheless, previous studies reported that IC_50_ values for purvalanol A on recombinant *Pf*PK5 and *Pf*mrk are respectively of 8 µM and 26 µM, suggesting the likely existence of alternative *in vivo* molecular targets [32,70].

With the marked exception of compound **20**, the screened molecules in the second round of assays exhibited significant IC_50_ values ranging from 0.7 µM to 7 µM on both strains (Table 2) and therefore appeared to be more potent than (*R*)-roscovitine and purvalanol A [71], and also more potent than other adenine and adenosine derivatives with a purine ring [72].

**Table 2:** IC_50_ values for *Pf*3D7 and *Pf*7G8 for the six mammalian kinase inhibitors and the three control molecules. These values were estimated by non-linear regression analysis using an inhibitory sigmoid *E*_max_ model. All assays were run in triplicates.

Compounds	Growth IC_50_ (µM)	
	*Pf*3D7	*Pf*7G8
**11**	6.53	2.06
**12**	5.37	1.55
**19**	7.13	1.82
**20**	22.21	4.66
**21**	3.05	0.69
**22**	1.39	0.99
(*R*)-roscovitine, **1**	10.48	12.56
Purvalanol A, **2a**	4.58	8.12
Chloroquine	0.03	0.18

Interestingly, these six inhibitors appeared to be 3- to 5-fold more potent against *Pf*7G8 than against *Pf*3D7 (e.g., compound **21**: *Pf*3D7 IC_50_ = 3.05 µM *vs.* *Pf*7G8 IC_50_ = 0.69 µM). Such marked sensitivity of the chloroquino-resistant strain has been frequently observed, and is generally due to an acute internalization of the drug into the protozoan. Indeed, protozoans such as *P. falciparum* are unable to synthesize purines *de novo*, and all their purines are provided by the host. To this end, these organisms have developed very effective and specific systems to take up and internalize purines. Thus, these uptake pathways might differ from one strain to another, and therefore might be the reason of these slight differences observed for *P. falciparum* growth inhibition. However, these differences observed between *P. falciparum* growth inhibition according to the chloroquine sensitivity could also depend on the specific PfCDK inhibited by the tested compounds which remain to be studied.

Compounds **11**, **12**, **21** and **22** which exhibited sub-micromolar IC_50_ for the mammalian kinases appeared to be promising inhibitors of *P. falciparum* growth with IC_50_ values around 1–5 µM for both strains. These results are probably correlated with the similar sequence and structure between mammalian and protozoan kinases. Nevertheless, further “hit to lead” improvement will be necessary to improve selectivity towards protozoan growth inhibition. To address this optimization, it might be interesting to consider molecules **18** and **22** IC_50_ values. Indeed, for these compounds, the protozoan growth inhibition potency is directly linked to the purine C^2^ pyridine core nitrogen position, suggesting a potential H-bond involving this nitrogen atom and the protozoan cellular target. This is a major difference with CDK inhibition, as we previously reported very close IC_50_ values for both of them on CDK1, CDK2 and CK1 (compound **18**: CDK1 : IC_50_ = 0.41 µM; CDK5: IC_50_ = 0.73 µM; CK1: IC_50_ = 0.10 µM; compound **22** : CDK1: IC_50_ = 0.59 µM; CDK5: IC_50_ = 0.60 µM; CK1: IC_50_ = 0.08 µM [44]). Starting from this, a specific anti-malaria pharmacophore introduction in another position of the purine scaffold might pave the way to the design of a more specific and potent “hit”.

## 3. Experimental Section

### 3.1. Chemistry

#### General Procedures

Chemical reagents and solvents were purchased from Sigma-Aldrich (Lyon, France), Fluka (Lyon, France) and Carlo Erba (Val de Reuil, France). Reactions were monitored by TLC using Merck (Fontenay sous Bois, France) silica gel 60F-254 thin layer plates. Column chromatographies were performed on SDS Chromagel 60 ACC 40‑63 µM. Melting points were determined on a Reichert Köfler hot-stage (Depew, NY, USA) and are uncorrected. NMR spectra were recorded on Bruker (Wissembourg, France) Avance 400 MHz (100 MHz for ^13^C-NMR) at 300 K. Chemical shifts were reported as δ values (ppm) indirectly referenced to the solvent signal or to tetramethylsilane (TMS) as internal standards. Data are reported in the conventional form. Mass spectra were recorded on a ZQ 2000 Waters using a Z-spray (ESI-MS).

*General Procedure for N^9^ alkylation.* To a solution of 2,6-dichloro-9*H*-purine or 2-amino-6-chloro-9*H*-purine (1.0 eq.) in DMSO (5.5 mL for 1 mmol of purine) at 15 °C were added K_2_CO_3_ (3.0 eq.) and 2-bromopropane (5.0 eq.). After 2–3 days stirring at 15–18 °C, water was added and the solution was extracted with EtOAc. The organic layer, once combined, was washed with brine, dried (Na_2_SO_4_), concentrated and purified by chromatography on silica gel columns (eluant: cyclohexane/EtOAc 1:9) to yield white solids (60%–75%).

2-Amino-6-chloro-9-iso-propylpurine (**7**) and 2,6-dichloro-9-*iso*-propylpurine (**9**) have been previously synthesized [44,72]; ^1^H-NMR, ^13^C-NRM and mass spectra are in accordance with previously reported data.

*General Procedure for Buchwald-Hartwig Amination*. A solution of Pd(OAc)_2_ and BINAP in dry toluene was warmed at 45 °C for 5 min. The leaving group-containing purine was then added under N_2_ bubbling; the mixture was kept at 45 °C for 10 min. and KOtBu was added. After 10 min, the appropriate nucleophile was added. The mixture was heated at 100 °C under N_2_ until reaction completion (3 h to 2 days depending upon the nucleophile used). After cooling to room temperature, the mixture was filtered through Celite, and concentrated. The residue was dissolved in CH_2_Cl_2_ (75 mL) and washed (1 × 10 mL) with water and brine (2 × 10 mL). The organic layer was dried and concentrated under vacuum. The residue was purified by chromatography on silica gel using various amounts of EtOAc/cyclohexane/ethanol as eluants.

We already reported chemical characterization for compounds **5**, **6** and **6'**; ^1^H-NMR, ^13^C-NMR and mass spectra are in accordance with previously reported data [44].

*2-Amino(pyrimidin-2-yl)-6-chloro-9-iso-propylpurine* (**8**). ^1^H-NMR (400 MHz, CDCl_3_): δ (ppm) 1.63 (d, 6H, *J* = 6.8 Hz, (C*H*_3_)_2_-CH), 4.87 (hept, 1H, *J* = 6.8 Hz, C*H*(CH_3_)_2_), 6.95 (t, 1H, *J* = 4.8 Hz, H_pyrimidinyl_), 8.00 (s, 1H, H-8_purine_), 8.31 (s, 1H, N*H*), 8.62 (d, 2H, *J* = 4.8 Hz, H_pyrimidinyl_).

*2-Amino(pyrimidin-2-yl)-6-[4-bromophenyl]-9-iso-propylpurine* (**4a**). ^1^H-NMR (400 MHz, CDCl_3_): δ (ppm) 1.62 (d, 6H, *J* = 6.8 Hz, (C*H*_3_)_2_-CH), 4.81 (hept, 1H, *J* = 6.8 Hz, C*H*(CH_3_)_2_), 6.95 (t, 1H, *J* = 4.8 Hz, H_pyrimidinyl_), 7.01 (m, 2H, *J* = 7.3 Hz, H_bromophenyl_), 7.34 (d, 2H, *J* = 7.3 Hz, H_bromophenyl_), 7.99 (s, 1H, H-8_purine_), 8.08 (m, 2H), 8.62 (d, 2H, *J* = 4.8 Hz, H_pyrimidinyl_); ^13^C-NMR (100 MHz, CDCl_3_) : δ (ppm) 21.4, 45.3, 114.9, 116.7, 120.4, 121.2, 133.4, 137.6, 147.9, 150.9, 151.3, 157.2, 157.3, 159.5; MS (ES^+^) *m/z* 425.08 [M+H]^+^.

*2-Amino(pyrimidin-2-yl)-6-[pyridin-2-yl]-9-iso-propylpurine* (**4b**). ^1^H-NMR (400 MHz, CDCl_3_): δ (ppm) 1.62 (d, 6H, *J* = 6.8 Hz, (C*H*_3_)_2_-CH), 4.80 (hept, 1H, *J* = 4.8 Hz, C*H*(CH_3_)_2_), 6.92 (t, 1H, *J* = 4.8 Hz, H_pyrimidinyl_), 6.99 (m, 1H, H_pyridyl_), 7.54 (m, 1H, H_pyridyl_), 7.83 (s, 1H, H-8_purine_), 8.12 (bs, 2H, N*H*), 8.32 (m, 1H, H_pyridyl_), 8.62 (d, 2H, *J* = 4.8 Hz, H_pyrimidinyl_), 9.29 (m, 1H, H_pyridyl_); ^13^C NMR (100 MHz, CDCl_3_) : δ (ppm) 22.4, 22.5, 45.3, 113.7, 114.9, 117.8, 120.4, 133.4, 137.6, 147.9, 149.4, 150.7, 151.2, 157.4, 158.0, 159.4; MS (ES^+^) *m/z* 348.08 [M+H]^+^.

*2-Amino(pyrimidin-2-yl)-6-[pyridin-3-yl]-9-iso-propylpurine* (**4c**). ^1^H-NMR (400 MHz, CDCl_3_): δ (ppm) 1.62 (d, 6H, *J* = 6.8 Hz, (C*H*_3_)_2_-CH), 4.81 (hept, 1H, *J* = 6.8 Hz, C*H*(CH_3_)_2_), 6.93 (t, 1H, *J* = 4.8 Hz, H_pyrimidinyl_), 7.30 (m, 1H, H_pyridyl_), 7.80 (s, 1H, H-8_purine_), 8.08 (s, 2H, N*H*), 8.32 (m, 1H, H_pyridyl_), 8.64 (d, 2H, *J* = 4.8 Hz, H_pyrimidinyl_), 8.86 (m, 1H, H_pyridyl_), 9.14 (s, 1H, H_pyridyl_). ^13^C-NMR (100 MHz, CDCl_3_): δ (ppm) 22.4, 45.3, 113.7, 114.6, 123.2, 126.7, 135.4, 136.4, 141.9, 142.9, 150.7, 151.2, 157.6, 158.1, 159.0; MS (ES^+^) *m/z* 348.14 [M+H]^+^.

*2-Amino(pyrimidin-2-yl)-6-[pyridin-4-yl]-9-iso-*propylpurine (**4d**). ^1^H-NMR (400 MHz, CDCl_3_): δ (ppm) 1.61 (d, 6H, *J* = 6.8 Hz, (C*H*_3_)_2_-CH), 4.82 (hept, 1H, *J* = 6.8 Hz, C*H*(CH_3_)_2_), 6.93 (t, 1H, *J* = 4.8 Hz, H_pyrimidinyl_), 7.76 (s, 1H, H-8_purine_), 7.69 (d, 2H, J = 6.0 Hz, H_pyridyl_), 8.00 (s, 2H, N*H*), 8.32 (d, 1H, *J* = 4.8 Hz), 8.64 (d, 2H, *J* = 4.8 Hz, H_pyrimidinyl_), 8.41 (d, 2H, *J* = 6.0 Hz, H_pyridyl_). ^13^C NMR (100 MHz, CDCl_3_): δ (ppm) 22.3, 46.1, 113.4, 115.5, 135.4, 136.4, 141.9, 142.9, 150.7, 151.2, 157.7, 158.1, 159.0.

*Chemical Characterizations of Compounds* **10**–**13**. These compounds have been obtained following a previously reported synthesis strategy. See references [43] and [46] for the details of corresponding experimental procedures and characterizations of intermediates.

*(R)-2-(1-Hydroxybut-2-ylamino)-6-[6-(3-pyridyl)pyrid-3-ylamino]-9-iso-propylpurine* (**10**). ^1^H-NMR (400 MHz, CDCl_3_): δ (ppm) 1.00 (t, 3H, *J* = 7.0 Hz, C*H*_3_CH_2_), 1,50 (d, 6H, *J* = 6.9 Hz, CH (C*H*_3_)_2_), 1.45–1.7 (m, 2H, C*H*_2_CH_3_), 3.5 (m, 1H, C*H*_2_OH), 3.8 (d, 1H, *J* = 3.1 Hz and *J* = 10.2 Hz, C*H*_2_OH), 3.9 (m, 1H, C*H*NH), 4.53 (hept, 1H, *J* = 6.9 Hz, C*H*(CH_3_)_2_), 7.3 (d, 1H, *J* = 8.0 Hz, H_pyridyl_), 7.55 (s, 1H, H-8_purine_), 7.7 (d, 1H, *J* = 8.0 Hz, H_pyridyl_), 7.75 (m, 2H, N*H*), 8.2–8.3 (m, 2H, H_pyridyl_), 8.55 (s, 1H, H_pyridyl_), 9 (d, 1H, *J* = 8.0 Hz, H_pyridyl_), 9,1 (s, 1H, H_pyridyl_). ^13^C-NMR (100 MHz, CDCl_3_) : δ (ppm) 10.8, 22.0, 23.1, 47.8, 55.9, 67.0, 120.8, 124.4, 134.5, 139.7, 140.3, 141.5, 147.9, 149.9, 150.8, 151.9, 157.4, 159.8

*(S)-2-(1-Hydroxybut-2-ylamino)-6-[3-(2-pyridyl)phenylamino]-9-iso-propylpurine* (**11**). ^1^H-NMR (400 MHz, CDCl_3_): δ (ppm) 0.98 (t, 3H, *J* = 7.2 Hz, C*H*_3_CH_2_), 1.49 (d, 6H, *J* = 6.8 Hz, CH(C*H*_3_)_2_), 1.67–1.78 (m, 2H, CH_3_C*H*_2_), 3.63 (m, 1H, C*H*_2_OH), 3.74 (dd, 1H, *J* = 2.4 Hz and *J* = 10.6 Hz, C*H*_2_OH), 3.97–4.05 (m, 1H, C*H*NH), 4.58 (hept, 1H, *J* = 6.8 Hz, C*H*(CH_3_)_2_), 4.94 (m, 1H, CHN*H*), 7.10–7.15 (m, 2H, H_pyridyl_), 7.38 (t, 1H, *J* = 7.2 Hz, H_phenyl_), 7.50–7.54 (m, 2H, H_phenyl_ + H_pyridyl_), 7.68–7.71 (m, 3H, H_phenyl_ + H_pyridyl_), 7.86 (s, 1H, H-8_purine_), 8.62 (d, 1H, *J* = 4.3 Hz, H_pyridyl_). ^13^C-NMR (100 MHz, CDCl_3_) : δ (ppm) 10.8, 22.5, 22.6, 24.9, 46.4, 55.9, 66.7, 114.9, 118.6, 120.3, 121.0, 121.4, 122.2, 129.1, 135.1, 136.9, 139.8, 140.1, 149.4, 152.2, 157.3, 159.6.

*(S)-2-(1-Hydroxybut-2-ylamino)-6-[3-(3-pyridyl)phenylamino]-9-iso-propylipurine* (**12**). ^1^H-NMR (400 MHz, CDCl_3_): δ (ppm) 0.94 (t, 3H, *J* = 6.8 Hz, C*H*_3_CH_2_), 1.49 (d, 6H, *J* = 6.8 Hz, CH(C*H*_3_)_2_), 1.53–1.66 (m, 2H, CH_3_C*H*_2_), 3.61 (m, 1H, C*H*_2_OH), 3.78 (m, 1H, C*H*_2_OH), 3.88–3.98 (m, 1H, C*H*NH), 4.56 (hept, 1H, *J* = 6.8 Hz, C*H*(CH_3_)_2_), 4.94 (d, 1H, *J* = 6.1 Hz, CHN*H*), 7.10–7.15 (m, 2H, H_pyridyl_), 7.29 (m, 1H, H_pyridyl_), 7.37 (t, 1H, *J* = 7.2 Hz, H_pyridyl_), 7.53 (s, 1H, H-8_purine_), 7.62–7.70 (m, 2H, H_phenyl_ + H_pyridyl_), 7.82 (d, 1H, *J* = 7.6 Hz, H_phenyl_), 7.99 (s, 1H, H_phenyl_), 8.52 (d, 1H, *J* = 4.1 Hz, H_pyridyl_), 8.81 (s, 1H, H_pyridyl_). ^13^C NMR (100 MHz, CDCl_3_) : δ (ppm) 10.8, 22.5, 24.7, 46.6, 56.0, 67.2, 115.1, 118.5, 119.5, 121.6, 123.5, 129.5, 134.5, 135.3, 136.5, 138.5, 139.8, 148.3, 148.5, 152.3, 159.6.

*(S)-2-(1-Hydroxybut-2-ylamino)-6-[4-(2-pyridyl)phenylamino]-9-iso-propylpurine* (**13**). ^1^H-NMR (400 MHz, CDCl_3_): δ (ppm) 0.98 (t, 3H, *J* = 7.2 Hz, C*H*_3_CH_2_), 1.46 (d, 6H, *J* = 6.8 Hz, CH(C*H*_3_)_2_), 1.52–1.68 (m, 2H, CH_3_C*H*_2_), 3.63 (dd, 1H, *J* = 6.7 Hz and *J‘* = 10.8 Hz, C*H*_2_OH), 3.81 (dd, 1H, *J* = 2.8 Hz and *J* = 10.8 Hz, C*H*_2_OH), 3.90–3.95 (m, 1H, C*H*NH), 4.53 (hept., 1H, *J* = 6.8 Hz, C*H*(CH_3_)_2_), 5.05 (m, 1H, CHN*H*), 7.10–7.15 (m, 2H, H_pyridyl_), 7.51 (s, 1H, H-8_purine_), 7.60–7.68 (m, 2H, H_pyridyl_), 7.80 (d, 2H, *J* = 8.4 Hz, H_phenyl_), 7.90 (d, 2H, *J* = 8.4 Hz, H_phenyl_), 8.59 (m, 1H, H_pyridyl_). ^13^C-NMR (100 MHz, CDCl_3_) : δ (ppm) 10.4, 21.8, 24.5, 45.8, 55.4, 65.8, 114.3, 119.5, 119.6, 121.1, 127.0, 132.7, 134.7, 136.4, 140.1, 148.7, 150.5, 151.4, 158.8, 159.7.

### 3.2. Biology—Protein Kinase Assays

#### 3.2.1. Biochemical Reagents

Sodium orthovanadate, EGTA, EDTA, MOPS, β-glycerophosphate, phenylphosphate, sodium fluoride, dithiothreitol (DTT), glutathione-agarose, glutathione, bovine serum albumin (BSA), nitrophenylphosphate, leupeptine, aprotinine, pepstatin, soybean trypsin inhibitor, benzamidine, and histone H1 (type III-S) were obtained from Sigma Chemicals (Lyon, France). [γ-^33^P]-ATP was obtained from Amersham (Pittsburgh, PA, USA). The CK-S peptide (RRKHAAIGpSAYSITA) (pS stands for phosphorylated serine) was purchased from Millegen (Labege, France), and the GS-1 peptide (YRRAAVPPSPSLSRHSSPHQpSEDEEE) was obtained from GenScript Corporation (Piscataway, NJ, USA).

#### 3.2.2. Buffers

Buffer A: 10 mM MgCl_2_, 1 mM EGTA, 1 mM DTT, 25 mM Tris-HCl pH 7.5, 50 µg heparin/mL. Buffer C: 60 mM β-glycerophosphate, 15 mM p-nitrophenylphosphate, 25 mM MOPS (pH 7.2), 5 mM EGTA, 15 mM MgCl_2_, 1 mM DTT, 1 mM sodium vanadate, 1 mM phenylphosphate.

#### 3.2.3. Kinase Preparations and Assays

Kinase activities were assayed in Buffer A or C, at 30 °C, at a final ATP concentration of 15 µM. Blank values were subtracted and activities expressed in % of the maximal activity, *i.e.*, in the absence of inhibitors. Controls were performed with appropriate dilutions of DMSO.

CDK1/cyclin B (M phase starfish oocytes, native), CDK2/cyclin A (human, recombinant) and CDK5/p25 (human, recombinant) were prepared as previously described [73]. Their kinase activity was assayed in buffer C, with 1 mg histone H1/mL, in the presence of 15 µM [γ-^33^P] ATP (3000 Ci/mmol; 10 mCi/mL) in a final volume of 30 µL. After 30 min incubation at 30 °C, 25 µL aliquots of supernatant were spotted onto 2.5 cm × 3 cm pieces of Whatman P81 phosphocellulose paper, and, 20 s later, the filters were washed five times (for at least 5 min each time) in a solution of 10 mL of phosphoric acid/liter of water. The wet filters were counted in the presence of 1 mL of ACS (Amersham, Pittsburgh, PA, USA) scintillation fluid.

GSK-3α/β (porcine brain, native) was assayed, as described for CDK1 but in Buffer A and using a GSK-3 specific substrate (GS-1: YRRAAVPPSPSLSRHSSPHQSpEDEEE) (pS stands for phosphorylated serine).

CK1δ/ε (porcine brain, native) was assayed in three-fold diluted buffer C, as described for CDK1 but using 25 µM CKS peptide (RRKHAAIGpSAYSITA), a CK1-specific substrate [74].

DYRK1A (human, recombinant, expressed in *E. coli* as a GST fusion protein) was purified by affinity chromatography on glutathione-agarose and assayed in buffer A (+0.5 mg BSA/mL) with using Woodtide (KKISGRLSPIMTEQ) (1.5 µg/assay) as a substrate.

### 3.3. Biology—In Vitro Drug Susceptibility Assays

Compounds were tested against synchronous ring-stage parasites of *Pf*3D7 and *Pf*7G8 strains. Drugs testing were carried out in 96-well microtiter plates. The chloroquine (CQ) diphosphate was purchased from Sigma (Lyon, France). The CQ was dissolved and diluted in water to obtain final concentrations ranging from 12.5 to 3,200 nM. The compounds were dissolved in DMSO and diluted in RPMI 1640 to obtain final concentrations ranging 0.1 µM to 100 µM. For each assay, each drug dilution was analyzed in triplicate.

For the *in vitro* isotopic microtest, 200 µL of the suspension of synchronous parasitized red blood cells with >90% of the parasites at the ring stage (final parasitaemia, 0.3%; final haematocrit, 4%) per well were plated in 96-well plates that contained serial CQ or compounds concentrations. Parasite growth was measured by the incorporation of radiolabeled [^3^H]hypoxanthine with a specific activity of 14.1 Ci/mmol (Perkin-Elmer, Courtaboeuf, France) (1 µCi per well) to each well at time zero. The plates were incubated at 37 °C, in an atmosphere of 5% O_2_, 5% CO_2_, and 90% N_2_ for 48 h. Immediately after incubation, plates were frozen and then thawed to lyse the erythrocytes. Cultures were harvested onto glass fiber filters and washed using a cell harvester (FilterMAT; Skatron Instruments, Dalsletta, Norway). The dried fiber filter papers were mixed with 2 mL of scintillation fluid (OptiScint; Perkin-Elmer, Waltham, UK). The radioactivity was counted using a liquid scintillation counter (Wallac 1410; Perkin-Elmer, Waltham, UK). The results were recorded as counts per minute (cpm) per well at each drug concentration. The drug concentration that could inhibit 50% of the parasite growth (IC_50_) were estimated by non-linear regression analysis using an inhibitory sigmoid *E*_max_ model (percent) = 100 − [(100 × *C*^γ^)/(*C*^γ^ + IC_50_^γ^)], where *C* corresponds to the drug concentration and γ is the sigmoidicity factor. The initial value and the asymptotic result for high concentrations were fixed to 0% and 100%, respectively [75], available on the web [76].

## 4. Conclusions

Using a convergent synthesis route which includes as a key step a Buchwald-Hartwig amination, we report the synthesis of new series of 2,6,9-trisubstituted purines structurally-related to (*R*)-roscovitine. The evaluation of these molecules as inhibitors on a mammalian kinases panel revealed that several compounds (**5**, **6**, **10** and **11**) exhibit marked activities against CDK5, CK1 and DYRK1A, a set of kinases involved in several neuronal pathologies such as Down Syndrome and Alzheimer’s disease. These molecules might be considered as starting “hits” for further structural optimization.

These newly-synthesized purines were also evaluated as potential inhibitors of *P. falciparum* growth *in vitro*. By means of a two-step screening assay, we identified six molecules which are more potent against the two *P. falciparum* clones (*Pf*3D7 and *Pf*7G8) than (*R*)-roscovitine and purvalanol A. By comparison with chloroquine, these growth inhibitions are still modest, but further structural optimizations of the purine scaffold are possible. Thus, further studies should allow us to determine whether potent anti-malarial motives might be introduced into the purine scaffold. Another key point to address will be the improvement of the compound selectivity, as our strongest *P. falciparum* growth inhibitors also appeared among the best mammalian CDK inhibitors we designed.

Altogether, these results open the way to further synthesis of new drugs targeting one of the most deadly disease in the world, malaria.

## Appendix

**Table A1:** IC_50_ values of the screened compounds on HsCDKs, and comparison with the *Plasmodium faciparum* 3D7 and 7G8 strains growth inhibition. Purines were tested at various concentrations as described in experimental section. IC_50_ values are reported in µM. On mammalian kinases, IC_50_ were estimated from dose-response curves and are expressed in µM. All assays were run in triplicates and data points were within less than a 10% range. -, not tested. IC_50_ value reported as >10 means that less than 50% inhibition at 10 µM was observed; N.I. (Not Inhibitor) indicates that the compound did not display any inhibitory activity. On *Pf* strains, IC_50_ were estimated by non-linear regression analysis using an inhibitory sigmoid *E*_max_ model. All assays were run in triplicates.

Compounds	IC_50_ on *P. falciparum* Strains (µM)		IC_50_ on HsCDKs (µM)	
	*Pf*3D7	*Pf*7G8	HsCDK1	HsCDK5
**1**	10.48	12.56	0.35	0.20
**2a**	4.58	8.12	0.004	0.075
**4b**	N.I.	N.I.	12	>10
**4c**	N.I.	N.I.	3.0	4.3
**10**	N.I.	N.I.	0.01	0.05
**11**	6.53	2.06	0.8	0.4
**12**	5.37	1.55	0.2	0.1
**13**	N.I.	N.I.	0.2	-
**14**	N.I.	N.I.	0.05	0.03
**15**	N.I.	N.I.	0.18	0.05
**16**	N.I.	N.I.	0.18	0.10
**17**	N.I.	N.I.	0.18	0.05
**18**	N.I.	N.I.	0.41	0.73
**19**	7.13	1.82	0.5	0.27
**20**	22.21	4.66	0.7	0.5
**21**	3.05	0.69	0.04	0.06
**22**	1.39	0.99	0.6	0.6
